# Identification and validation of basement membrane‐associated gene AGRN as prognostic and immune‐associated biomarkers in colorectal cancer patients

**DOI:** 10.1111/jcmm.70010

**Published:** 2024-08-25

**Authors:** Jianrong Li, Daofeng You, Linjie Hu, Yusi Yang, Sheng Gao, Wenqi Bai

**Affiliations:** ^1^ Department of General Surgery Sciences, Shanxi Province Cancer Hospital/Shanxi Hospital Affiliated to Cancer Hospital Chinese Academy of Medical Sciences/Cancer Hospital Affiliated to Shanxi Medical University Taiyuan China; ^2^ Emergency Department of First Hospital Affiliated to Hebei Medical University Shijiazhuang China; ^3^ Third Hospital of Shanxi Medical University, Shanxi Bethune Hospital, Shanxi Academy of Medical Sciences Tongji Shanxi Hospital Taiyuan China

**Keywords:** AGRN, basement membrane, colorectal cancer, differentially expressed genes, immune

## Abstract

Colorectal cancer (COCA) has a poor prognosis, with growing evidence implicating basement membranes (BMs) in cancer progression. Our goal was to investigate the role and predictive significance of BMs in COCA patients. We obtained BMs‐related genes from cutting‐edge research and used TCGA and GTEx databases for mRNA expression and patient information. Cox regression and LASSO regression were used for prognostic gene selection and risk model construction. We compared prognosis using Kaplan–Meier analysis and examined drug sensitivity differences. The CMAP dataset identified potential small molecule drugs. In vitro tests involved suppressing a crucial gene to observe its impact on tumour metastasis. We developed a 12 BMs‐based approach, finding it to be an independent prognostic factor. Functional analysis showed BMs concentrated in cancer‐associated pathways, correlating with immune cell infiltration and immune checkpoint activation. High‐risk individuals exhibited increased drug sensitivity. AGRN levels were linked to decreased progression‐free survival (*p* < 0.001). AGRN knockdown suppressed tumour growth and metastasis. Our study offers new perspectives on BMs in COCA, concluding that AGRN is a dependable biomarker for patient survival and prognosis.

## 1 INTRODUCTION

Colorectal cancer ranks as the third most common cancer globally, trailing behind lung cancer, and has demonstrated a rising incidence on a global scale in recent years.^1^ The early stages of colorectal cancer frequently present with subtle or absent symptoms, resulting in a significant proportion of cases being diagnosed at advanced stages. Despite notable progress in targeted and immune‐based therapies, the prognosis for individuals with advanced colorectal cancer remains unsatisfactory.^2^ Therefore, the development of novel predictive models and investigation of potential markers for disease progression are imperative in advancing the management of colorectal cancer.

The basement membrane, located on the basal surface of endothelial and epithelial cells, is a vital extracellular matrix component essential for tissue and organ development and function. Comprised of laminin and collagen IV, the basement membrane provides structural support, forms selective barriers and aids in signal transduction.^3^, ^4^, ^5^ Additionally, nitrogen sources, proteoglycans and growth factors are also present in this matrix.^6^ Recent studies have shown a correlation between genes related to the basement membrane and the prognosis of certain cancers, such as lung adenocarcinoma.^7^, ^8^, ^9^ In liver cancer, models of BM‐associated genes including MMP1, ITGA2, P3H1 and CTSA have been shown to accurately predict patient prognosis. A novel BM‐score gene signature for breast cancer patients can predict clinical prognosis and metastasis accurately, which may help with individualized clinical decision‐making. Additionally, the epithelial mesenchymal transition (EMT) of the basement membrane facilitates the spread of cancer from its original location to distant organs.

The association between the prognosis of COCA samples and these genes regulated by the basement membrane remains uncertain. For this study, we utilized the Cancer Genome Atlas (TCGA) repositories to collect gene expression data and details on individuals with COCA. Subsequently, a predictive multigene signature was developed using differentially expressed genes (DEGs) regulated by the basement membrane, which was validated through qRT‐PCR. Additionally, we discovered possible main controllers and confirmed the fundamental process through experimentation. Ultimately, we employed functional enrichment analysis to examine the primary procedures. Supplementary file S1 contains a summary of the study's design and key discoveries.

## 2 METHODS

### 2.1 Data gathering and the identification of differentially expressed BM‐related genes

Data on mRNA levels and clinical details for 41 healthy bladder samples and 480 COCA samples were retrieved from TCGA public database at https//portal.gdc.cancer.gov. Previous research on the topic yielded a collection of 225 genes related to BM. We also downloaded genote‐Tissue Expression (GTEx) datasets consisting of 308 normal colon tissues for validation.^10^ Normalization of these mRNA expression profiles was performed using the normalizeBetweenArrays R package. limma package was used to identify DEGs meeting the criteria (|logFC| >1 and FDR <0.05).

### 2.2 Protein–protein interactions and functional enrichment analysis

The R package ClusterProfiler was used for conducting Gene Ontology (GO), which included examining analysis, encompassing the examination of biological processes (BP), molecular functions (MF) and cellular components (CC). The Pathway analysis of pathways in the Kyoto Encyclopedia of Genes and Genomes (KEGG) was performed using an identical approach carried out using a similar methodology.^11^ The significance of enrichment was determined based on false discovery rate (FDR) and *p*‐values less than 0.05. The variously expressed below 0.05. Differentially expressed biological molecules (BMs) were sent in the STRING database (http://www.string‐db.org/) for protein–protein interaction (PPI) data obtained from high‐throughput experimental data, computerized genome prediction, automated text mining and other database data. Minimum required interaction score was set medium confidence (0.4). Cytoscape was used to build and show the PPI network. The MCODE plug‐in was utilized to identify the most important module in the PPI network based on MCODE scores greater than 10.

### 2.3 Screening for potential small molecule drugs

Various genes associated with bone metastases (BMs) were analysed using the Connectivity Map database (CMAP, https://portals.broadinstitute.org/cmap/) in order to identify potential small molecule drugs for the treatment of patients with cancer of unknown primary origin (COCA). Ratings on a scale from −1 to 1 were assigned to assess the correlation between the uploaded genes and the compounds in the database. Compounds with lower ratings may possess the potential to inhibit cancer cell proliferation. The predetermined criteria for significance were set at a *p*‐value of less than 0.01, a minimum sample size of 3, a non‐null percentage of 100, and an enrichment value below −0.8.

### 2.4 Building and verifying a BMs prognostic model

Our study conducted univariate Cox regression analysis to assess the prognostic relevance of genes associated with BMs. Subsequently, a penalized Cox regression analysis was employed to construct a prognostic risk model utilizing the glmnet R package, which facilitated the computation of risk scores.

The risk score is calculated by multiplying Coef1 by the expression of mRNA1.

$$\text{Risk}\;\text{score}=\left(\text{Coef1}\times \text{expression mRNA1}\right)+\left(\text{Coef2}\times \text{expression mRNA2}\right)+\left(\text{Coef}\;\text{n}\times \text{expression mRNA}\;\text{n}\right).$$

Coef represents the coefficient of the lasso Cox regression model for the mRNA in question. Based on the median of their risk scores, patients from the COCA trial were divided into high‐risk and low‐risk groups. Prognosis in two groups was evaluated through survival analysis using Kaplan–Meier curve. Using the survival ROC package, time‐related ROC analysis was used to evaluate the risk model's prognostic potential.

### 2.5 The process of Gene Set Enrichment Analysis

Gene Set Enrichment Analysis (GSEA) was conducted to investigate the molecular pathways associated with low‐risk and high‐risk groups. Statistical significance was defined as *p* values below 0.05 and false discovery rates (FDR) below 25%.

### 2.6 The creation of a nomogram using clinical factors and risk scores

The study centered on examining the relationship between a signature derived from BM and clinical factors. Additionally, univariate and multivariate Cox regression analyses were performed, alongside other clinical variables, to determine if risk scores could predict COCA patient prognosis. A nomogram was created to forecast the 3‐ and 5‐year overall survival rates in COCA patients using both clinical factors and the risk score derived from the BM‐based signature. The predictive accuracy of the nomogram was evaluated through calibration curve analysis and calculation of the concordance index (C‐index).

### 2.7 Immune cell infiltration analysis

Increasing evidence has shown that the presence of immune cells within tumour cells plays a role in the advancement of cancer and is linked to the outlook of the disease. As a result, we used a variety of algorithms, including XCELL, CIBERSORT, QUANTISEQ, MCP‐counter, CIBERSORT‐ABS, TIMER and EPIC, to determine the immune cell infiltration levels in the high‐risk and low‐risk groups. To anticipate the impact of immune checkpoint blockade treatment, we investigated the levels of various immune checkpoints including ADORA2A, CD48, CD80, CD86, CD200R1, CD274 and HAVCR2.Furthermore, we utilized the TIMER database to explore the connection between immune cells and 12 biomarkers, enhancing our comprehension of the involvement of biomarkers in COCA. Emerging research suggests that immune cell infiltration within tumour cells significantly influences cancer progression and prognosis.

### 2.8 CCK‐8 assay

Following a 24‐h transfection period with siRNA‐NC or siAGRN, SW480 cells were seeded onto 96‐well culture plates (6 × 10^9^/well). Add 200 μL of DMEM medium to each well. A Cell Counting Kit‐8 was used to quantify the proliferation of the cells at 0, 24, 48 and 72 h. After adding 20 μL of CCK8 solution to each well, the cells were incubated at 37°C for 2 h. The enzyme standard instrument is finally utilized to measure the absorbance of each well at a wavelength of 450 nm.Among them, siRNA‐NC was used as the control group.

### 2.9 Assays for cell invasion and migration

We set two groups: control group (si‐NC) and knockdown group (siAGRN). The three holes were established by each group. Transwell chambers (Corning, Kennebunk, ME, USA) were seeded with FBS‐free cell suspension at a density of 1–2 × 10^4^/well for the invasive experiment, Transwell Chambers (Corning, Kennebunk, ME, USA) were used with a cell suspension density of 2 × 10^4^ cells per hole without FBS for vaccination. In the migration experiment, the cell suspension density was adjusted to 1×10^4^ cells per hole without FBS for inoculation.

20% FBS was added to the supplemented medium in the bottom well. Cells on both sides of the membrane were incubated for 48 h, fixed for 20 min with 4% paraformaldehyde, and then exposed to the crystal violet dye for an additional 10 min. Using a cotton swab, the cells in the top chamber were carefully removed. Under a microscope, colorectal cells were counted and photographed.

### 2.10 SiRNA interference assay

We acquired AGRN siRNA and scrambled siRNA from IBSBIO (Shanghai, China). As a comparison, a scrambled siRNA was used. Following the manufacturer's instructions, Lipofectamine 3000 (Invitrogen) was used to transfect the siRNAs into colorectal cancer cells. 48 h after transfection, total RNA was collected, and 72 h later, protein. Western blot and quantitative RT‐PCR were used to measure the amounts of protein and RNA, respectively. The following were the siRNA sequences: AGAACGAGCUGAUGCUCAACU is the siAGRN‐1, CCUUUGUCGAGUACCUCAACGCUGU is the siAGRN‐2, and UUCUCCGAACGUGUCACGUTT is the siRNA‐NC (Table 1). siRNA‐NC was used as the control group, and siAGRN‐1 and siAGRN‐2 were used as knockdown groups.

**TABLE 1:** siRNA sequences for knockdown AGRN.

siRNA	siRNA sequences
AGRN‐384	Sense: 5′‐AGAACGAGCUGAUGCUCAACU‐3′
	Anti‐sense:5′‐UUGAGCAUCAGCUCGUUCUUG‐3′
AGRN‐5618	Sense: 5′‐CCUUUGUCGAGUACCUCAACGCUGU‐3′
	Anti‐sense: 5′‐ACAGCGUUGAGGUACUCGACAAAGG‐3′
siRNA‐NC	Sense: 5′‐UUCUCCGAACGUGUCACGUTT‐3′
	Anti‐sense: 5′‐UUUCUCUUUAGCUUCUUCCAG‐3′

### 2.11 Cell lines and cell culture

The normal human colonic epithelial cell line NCM460 was donated by Shanhai Beiweimin technology Co., Ltd. For the colorectal cell line SW480, Procell Life Science & Technology Co., Ltd. gave it.HT‐29 colorectal cell line from Abbkine Scientific Co., Ltd.NCM460 and SW480 cells were cultured in Dulbecco's Eagle's medium, which was adjusted with 1% penicillin–streptomycin and 10% (v/v) foetal bovine serum (Gibco). In McCoy'5a medium supplemented with 10% (v/v) FBS and 1% (P/S), HT‐29 cells were cultivated. These cell lines were kept in an environment with 5% CO_2_ at 37°C.

### 2.12 Analyses using the quantitative real‐time PCR

Polymerase chain reaction Colorectal cells were treated with the TransZol Up Plus RNA Kit to extract total RNA. Total RNA concentration and purity were measured using a Thermo Fisher Scientific, Wilmington, DE, USA, NanoDrop 2000 spectrophotometer. Complementary DNA was produced using the MonScriptTM RTllll Super Mix with dsDNase kit (Monad). Using MonAmpTM ChemoHS qPCR Master Mix, quantitative real‐time PCR was conducted under the following conditions: After 10 min at 95°C, 40 cycles of 10 s at 95°C and 30 s at 60°C were conducted. The primer sequences for detection are shown in Table 2. A standard control used internally was GAPDH expression. All gene expression levels were collected and quantified using the 2^−△△Ct^ method. The results came from three different trials. In this experiment, each group is set with three holes.

**TABLE 2:** primers for qRT‐PCR.

Gene name	Sequence (5′–3′)
GAPDH	F: 5′‐TGACTTCAACAGCGACACCCA‐3′ R: 5′‐CACCCTGTTGCTGTAGCCAAA‐3′
AGRN	F:5′‐GTCCTGCGTCTGCAAGAAGAG‐3′ R: 5′‐CACCCTGTTGCTGTAGCCAAA‐3′

### 2.13 Western blotting

Using western blot (WB) lysis buffer (BMP2020, Abbkine) containing PMSF, the protein was extracted, and a BCA kit (SW101‐02, Severn Biotech) was used for quantification. Protein extracts in an equivalent quantity (20 μg) were separated on 10% SDS‐PAGE gels and then transferred to PVDF membranes (Immobilon‐P, Millipore). After being blocked for 1.5 h in 5% non‐fat milk, the cell membrane was treated with the corresponding starting antibodies at 4°C for an entire night. The membranes were incubated with the secondary antibody (1: 5000 dilution; Biosharp, China) for 2 h at room temperature after being washed three times with TBST for 10 min. The signals were visualized using ECL reagent (BMU102‐CN, Abbkine, China) and quantified using Image Lab Software (Bio‐Rad) after a 30‐min TBST wash. AGRN (sc‐374,117, Santa Cruz), NOTCH1 (20687‐1‐AP, Proteintech), RBPJ (66132‐1‐Ig, Proteintech), HES1 (sc‐166,410, Santa Cruz) and GAPDH (BM1623, Boster) were the mentioned primary antibodies.

### 2.14 Statistical analyses

R software (version 4.0.5) was used for all statistical analyses. Differences between the two groups were compared using the Wilcoxon test. A *p*‐value less than 0.05 indicated statistical significance.

### 2.15 Mutation analyses

RNAseq data, mutational maf data, and corresponding clinical information were obtained from the Cancer Genome Atlas (TCGA) dataset (https://portal.gdc.com) for colorectal tumors. Use the maftools package in R software to download and visualize somatic mutations in patients with colorectal cancer. The horizontal histogram shows a higher frequency of mutations in colorectal cancer patients.

## 3 RESULTS

### 3.1 Establishment and validation of BMs‐based signature

**FIGURE 1:**
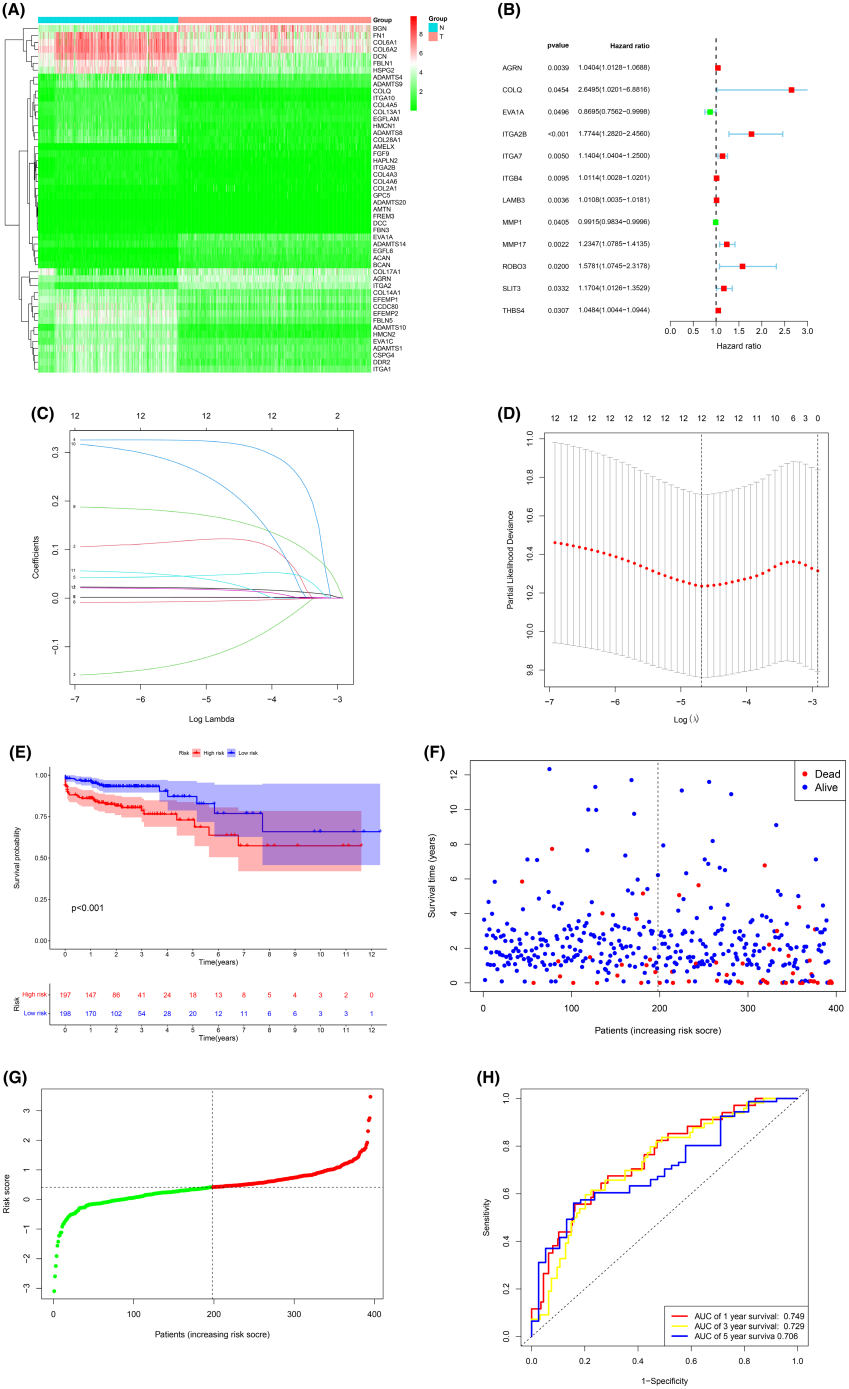
(A) Heatmap showed differentially expressed BMs. (B) Identification of prognostic BMs by univariate Cox regression analysis. (C) Plot of LASSO regression coefficients for variables. (D) Plot of LASSO ten‐fold cross‐validation results. (E) Kaplan–Meier survival analysis of COCA patients between high‐risk groups and low‐risk groups. (F, G). Distribution of survival status based on the median risk score. (H) Heatmap showed the differences of 12 BM‐related genes between high and low‐risk patients.

A comprehensive analysis was conducted on a combined dataset of 349 normal samples and 480 tumour samples, which included 308 typical colorectal tissues from the Genotype‐Tissue Expression (GTEx) database and 41 normal and 480 tumour samples from The Cancer Genome Atlas (TCGA) database. In comparison to healthy tissues, 108 genes associated with BMs^12^ exhibited differential expression levels in the combined dataset, with 75 genes showing down‐regulation and 33 genes showing upregulation (Figure 1A). The details of the difference analysis are written in supplementary file S2. The merged dataset was utilized to perform univariate Cox regression analysis to examine the prognostic relevance of genes linked to BMs. The results revealed that only 12 genes demonstrated predictive significance (Figure 1B). Subsequently, a LASSO Cox regression analysis was employed to formulate a prognostic signature for forecasting outcomes in colorectal cancer patients (Figure 1C,D). A successful construction of a risk model was achieved using 12 genes (AGRN, COLQ, EVA1A, ITGA2B, ITGA7, ITGB4, LAMB3, MMP1, MMP17, ROBO3, SLIT3 and THBS4).The risk score was determined by the coefficients of 12 CRs in the formula (Table 3): risk score = (0.0195 × AGRN expression) + (0.1221 × COLQ expression) + (−0.1076 × EVA1A expression) + (0.3199 × ITGA2B expression) + (0.0484 × ITGA7 expression) + (0.0019 × ITGB4 expression) + (0.0016 × LAMB3 expression) + (−0.0056 × MMP1 expression) + (0.1627 × MMP17 expression) + (0.2245 × ROBO3 expression) + (0.0314 × SLIT3 expression) + (0.0166 × THBS4 expression). Patients diagnosed with colorectal cancer were stratified into high‐risk and low‐risk categories using the median risk score as a cutoff point (see Figure 1F,G). The high‐risk group exhibited a significantly higher mortality rate compared to the low‐risk group (*p* < 0.001), suggesting an inverse relationship between risk score and prognosis (see Figure 1E). The receiver operating characteristic (ROC) analysis, which demonstrated temporal variability, revealed a predictive accuracy of 0.749 for the BMs‐derived signature after 1 year (Figure 1H).

**TABLE 3:** Gene list and coefcient.

Id	Coef
AGRN	0.0195
COLQ	0.1221
EVA1A	−0.1076
ITGA2B	0.3199
ITGA7	0.0484
ITGB4	0.0019
LAMB3	0.0016
MMP1	−0.0055
MMP17	0.1627
ROBO3	0.2245
SLIT3	0.0314
THBS4	0.0166

**FIGURE 2:**
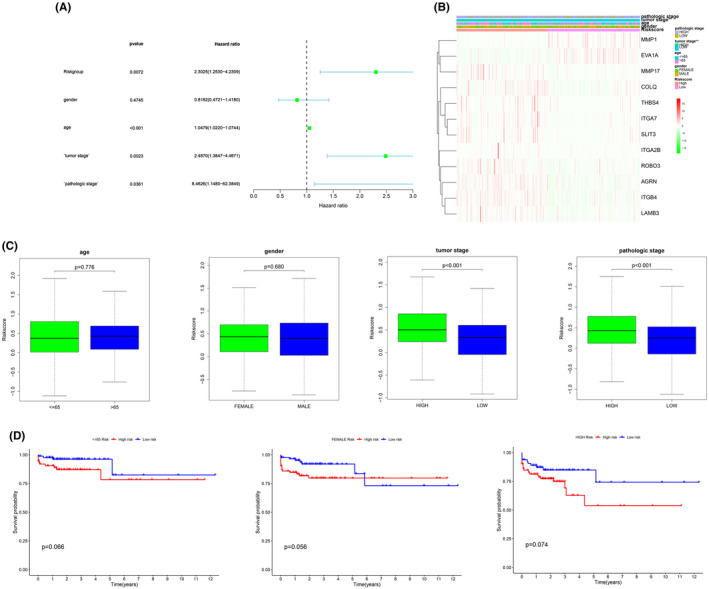
(A) The correlations between the risk score for OS and clinicopathological factors by univariate and multivariate cox regression analysis. (B, C). Correlation between signature and clinical characteristics. (D) Kaplan–Meier curves of OS differences stratified by gender, age, grade, N stage, T stage or TNM stage between the high‐risk groups and low‐risk groups.

The study utilized univariable and multivariable Cox analyses to assess the potential of the BMs‐based signature as an independent prognostic indicator for colorectal cancer. Univariate analysis revealed a significant association between patient survival and factors such as risk score, age, pathologic stage and tumour stage (*p* < 0.001) (Figure 2A), indicating that the BMs‐based signature holds promise as an independent prognostic indicator for colorectal cancer patients.

### 3.2 Association between the signature and clinical characteristics

The chi‐squared test was utilized to investigate if the prognostic signature played a role in the advancement and growth of colorectal cancer. Figure 2B,C displayed significant variances in tumour stage and pathologic stage (*p* < 0.001) between high‐ and low‐risk groups, with no notable distinctions in age or gender. Additionally, stratification analysis was performed to explore the prognostic importance of the signature within different subgroups. The chi‐square test was employed to examine the potential impact of the prognostic signature on the progression and development of colorectal cancer. Significant differences in tumour stage and pathologic stage (*p* < 0.001) were observed between high‐ and low‐risk groups, as shown in Figure 2B,C, with no significant variations in age or gender. Furthermore, stratification analysis was conducted to investigate the prognostic significance of the signature across various subgroups. The findings of our research suggest that a signature derived from bone marrow cells demonstrated high accuracy in predicting tumour stage (*p* < 0.001) and pathologic stage (*p* < 0.001) outcomes, as illustrated in Figure 2D. However, the predictive success of the BM‐based signature was found to be limited in relation to age and grade outcomes (*p* > 0.05).

### 3.3 Functional enrichment analyses and protein–protein interaction

**FIGURE 3:**
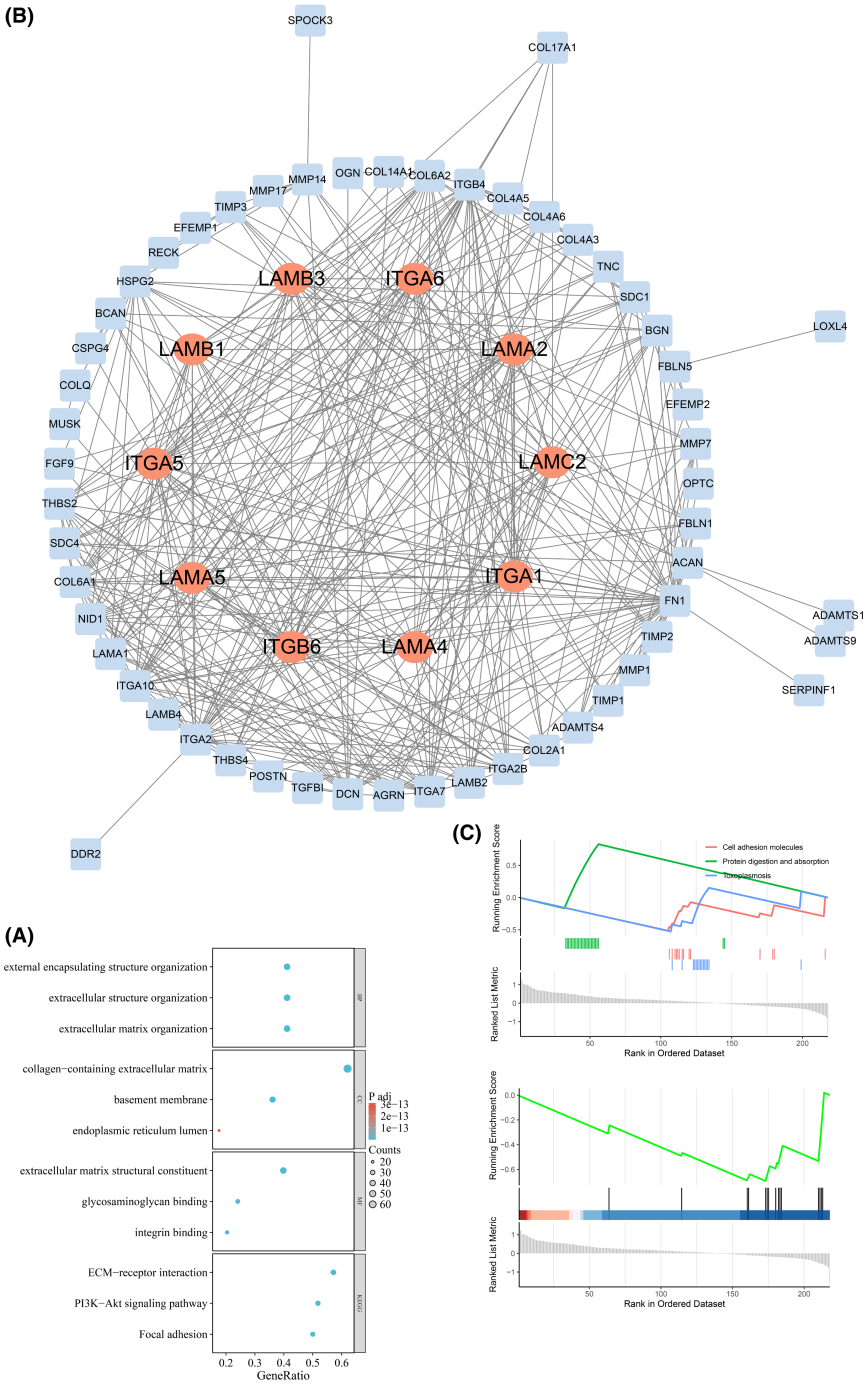
(A) Protein–protein interaction (PPI) network of differentially expressed BMs. (B) Enrichment analyses of differentially expressed BMs. (C) GSEA analysis.

The investigation of the potential roles of differentially expressed genes related to basement membranes was carried out using Gene Ontology (GO) and Kyoto Encyclopedia of Genes and Genomes (KEGG) analyses. The biological process analyses indicated that 108 genes associated with basement membranes were significantly involved in the organization of the extracellular matrix, extracellular structure and external encapsulating structure, as shown in Figure 3A.Examination of cellular constituents demonstrated a notable augmentation of collagen‐containing extracellular matrix, basement membrane and protein complexes pertinent to cell adhesion. Evaluation of molecular functions elucidated that 108 genes implicated in BMs were predominantly linked to extracellular matrix structural elements and glycosaminoglycan binding. The KEGG pathways analysis revealed that the identified genes were predominantly involved in ECM‐receptor interaction, PI3K‐Akt signalling pathway, and Focal adhesion. Furthermore, the PPI network of the genes associated with differentially expressed biomarkers was visualized in Figure 3B as indicated by the STRING database.

### 3.4 GSEA

In order to provide a more comprehensive understanding of the molecular mechanisms underlying the signature of biomarkers, GSEA was conducted. The results of the GSEA analysis revealed that the high‐risk group exhibited significant enrichment in the Protein digestion and absorption pathway, while the low‐risk group demonstrated enrichment in Cell adhesion molecules and Toxoplasmosis (Figure 3C).

### 3.5 Immune infiltration level analysis of the BMs‐based signature

**FIGURE 4:**
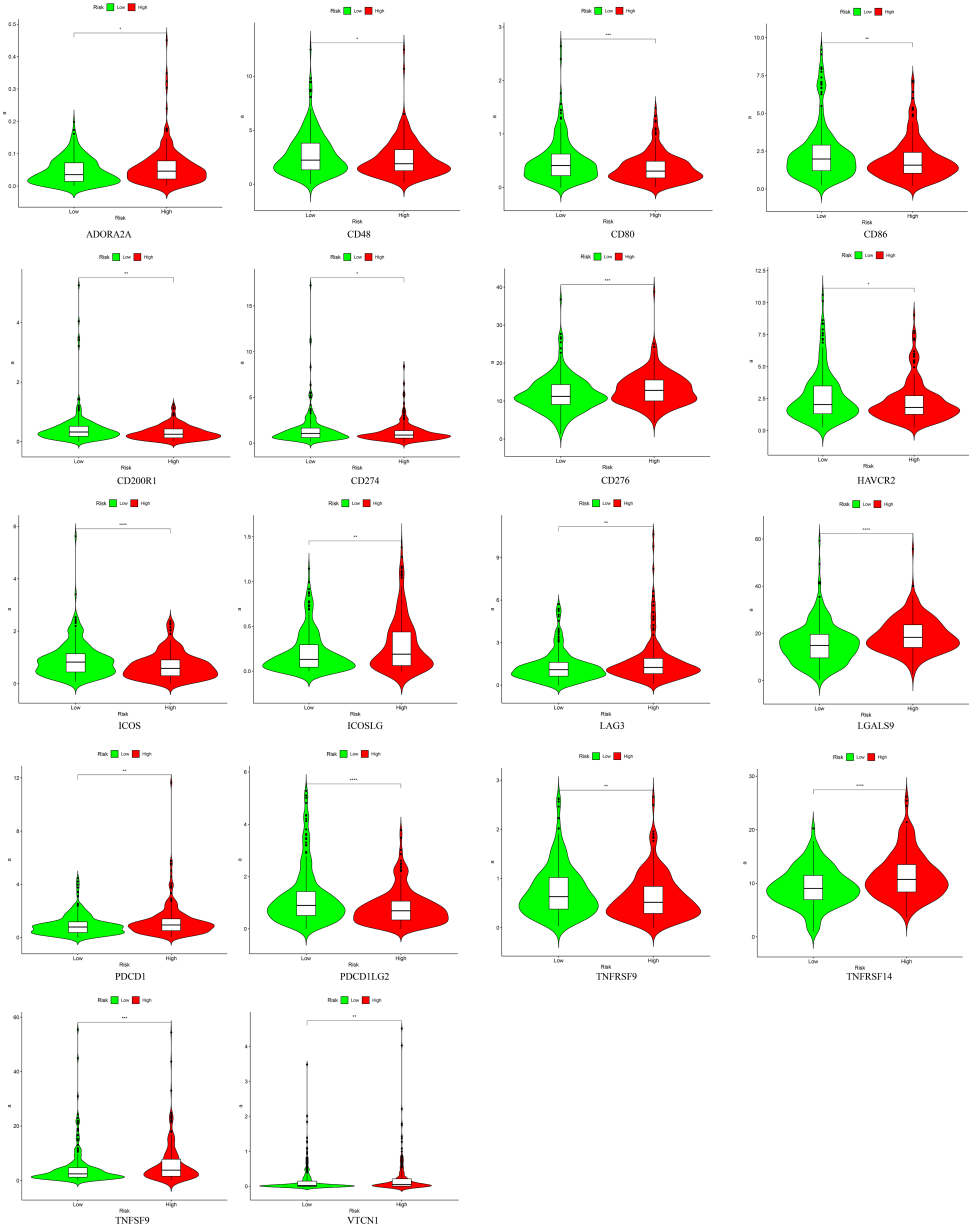
The relationship between prognostic signature and immune checkpoints.

The heatmap depicted the relationship between the signature and immune cell infiltration as determined by various computational tools, including TIMER, CIBERSORT, CIBERSORTABS, XCELL, QUANTISEQ, EPIC and MCP‐counter (see Supplementary file). Specifically, CIBERSORT analysis revealed elevated proportions of CD8+ T cells, Tregs, and CD4+ T cells in the high‐risk group. In light of the significant role of checkpoint inhibitor immunotherapies, we conducted a comprehensive analysis of the correlation between risk score and key immune checkpoints, such as ADORA2A, CD48, CD80, CD86, CD200R1, CD274, HAVCR2, ICOS, ICOSLG, LAG3, LGALS9, PDCD1, PDCD1LG2, TNFRSF9, TNFRSF14, TNFSF9 and VTCN1 (Figure 4). A notable disparity was observed in the levels of ADORA2A, CD48, CD80, CD86, CD200R1, CD274, HAVCR2, ICOS, ICOSLG, LAG3, LGALS9, PDCD1, PDCD1LG2, TNFRSF9, TNFRSF14, TNFSF9 and VTCN1 among the patient cohorts. Additionally, ADORA2A, CD276, ICOSLG, LAG3, LGALS9, PDCD1, TNFRSF14, TNFSF9 and VTCN1 exhibited elevated levels in the high‐risk groups, suggesting an immunosuppressed and fatigued phenotype in those populations.

### 3.6 Identification of small molecule drugs

Utilizing the CMAP database, we identified the top seven small molecule drugs based on their biological mechanisms. These drugs include ondansetron, anabasine, dimethylprostaglandin E2, idoxuridine, flfluvastatin, piribedil and trichlormethiazide, as outlined in Table 4.

**TABLE 4:** The 7 small molecule drugs of CMP dataset analyses results.

CMAP names	*n*	*p*‐value	Adjusted *p*‐value
Ondansetron	6	1.70258011951738E‐5	0.041169
Anabasine	5	2.0956816622055144E‐4	0.041169
Dimethylprostaglandin E2	5	2.0956816622055144E‐4	0.041169
Idoxuridine	5	2.0956816622055144E‐4	0.041169
Fluvastatin	5	2.0956816622055144E‐4	0.041169
Piribedil	5	2.0956816622055144E‐4	0.041169
Trichlormethiazide	5	2.0956816622055144E‐4	0.041169

### 3.7 Analysing the occurrence of genetic mutations and changes in chromosomal copy numbers among individuals with COCA

**FIGURE 5:**
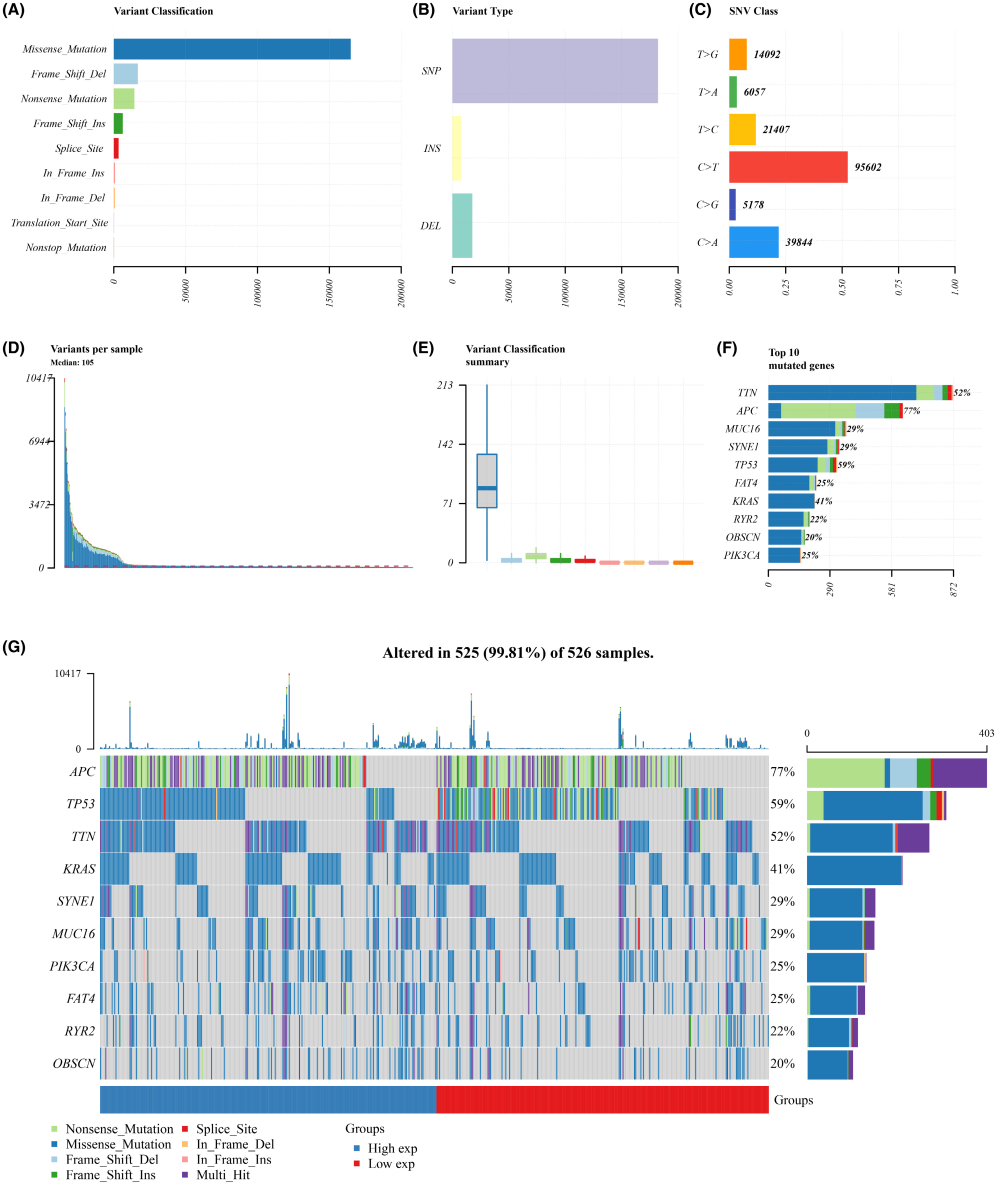
(A–F) Mutation panorama analysis. (G) Top 10 gene mutation waterfall map.

The mutation data of COCA patients was analysed to assess the impact of mutations on patient outcomes, with samples from the TCGA dataset lacking sufficient information being excluded. Our initial investigation revealed that SNPs were the predominant type of variation, with missense mutations comprising the majority of mutations identified in COCA patients. Moreover, our study revealed that the predominant single nucleotide polymorphism (SNP) in patients with COCA was the C > T variant (Figure 5A–C). Each patient's tally of base changes was computed, and distinct colours in the box plot were utilized to differentiate between various mutation types (Figure 5D,E). The findings suggest that TTN (52%), APC (77%), MUC16 (29%), SYNE1 (29%), TP53 (59%), FAT4 (25%), KRAS (41%), RYR2 (22%), OBSCN (20%) and PIK3CA (25%) were the top ten genes exhibiting the highest mutation frequencies in COCA. A detailed enumeration of the top ten mutated genes identified in each patient is presented in Figure 5G.

### 3.8 Analysis of the survival rates of the 12 genes in COCA

**FIGURE 6:**
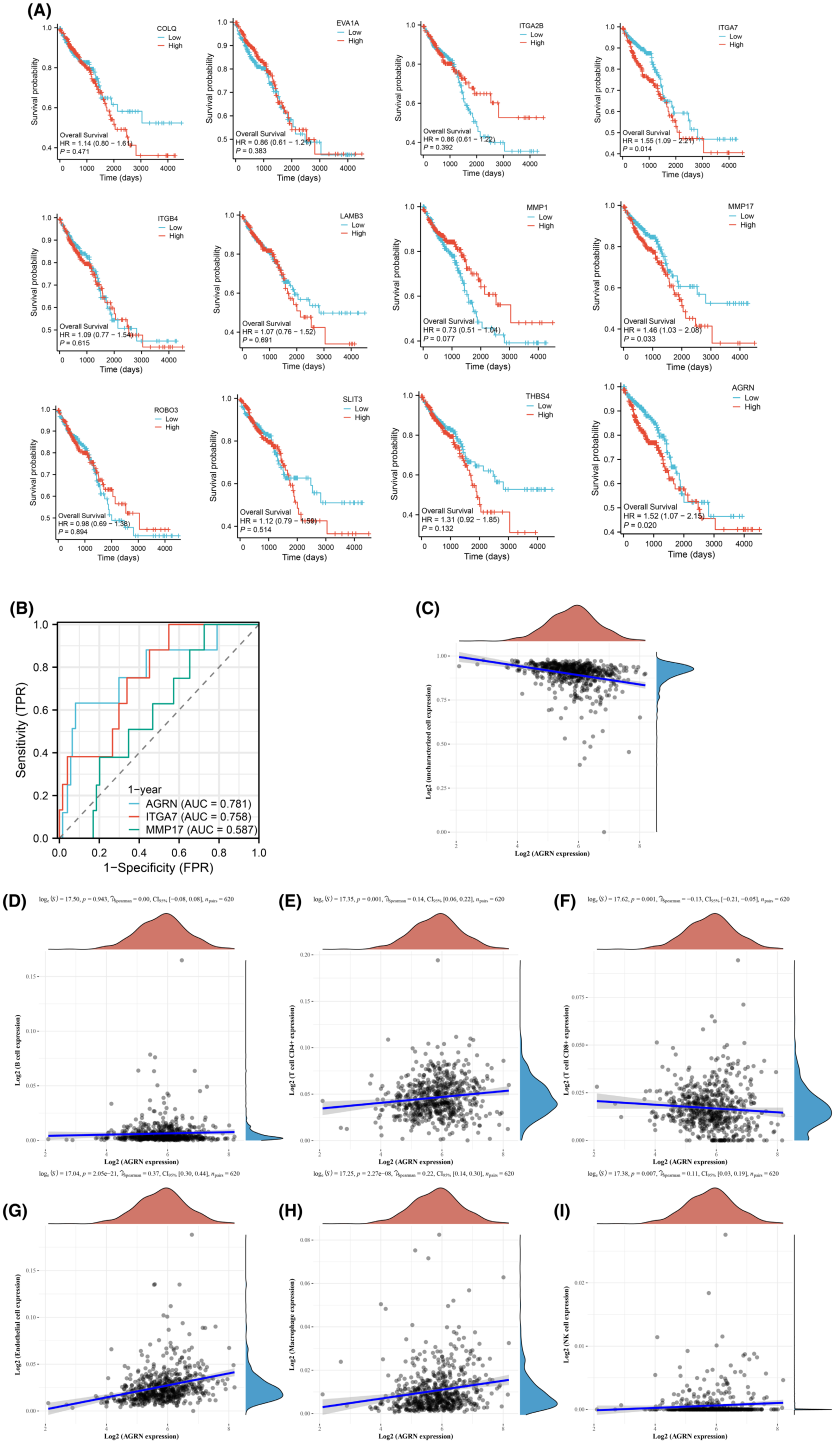
(A) OS of the 12 genes in patients with COCA was analysed by Kaplan–Meier plotter. (B) ROC plots of AGRN, ITGA7 and MMP17. (C–I). The relationship between immune cells and expression level of AGRN.

The 12 genes (AGRN, COLQ, EVA1A, ITGA2B, ITGA7, ITGB4, LAMB3, MMP1, MMP17, ROBO3, SLIT3 and THBS4) utilized in the model were subjected to OS analyses using the Kaplan–Meier plotter. The findings presented in Figure 6A indicate that heightened levels of AGRN, ITGA7 and MMP17 in COCA patients were associated with a detrimental effect on overall survival. Additionally, the ROC analysis of the remaining genes revealed that AGRN exhibited a more favourable 1‐year prognosis in COCA patients (Figure 6B). A Spearman correlation analysis was performed to investigate the potential role of AGRN in relation to immune score, as depicted in Figure 6C–I.

### 3.9 Identification of AGRN critical role in COCA

**FIGURE 7:**
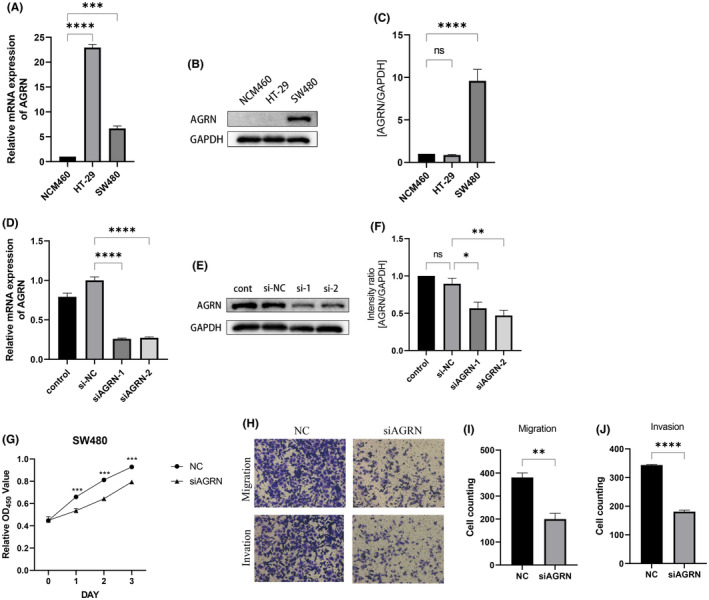
(A) Expression level of AGRN in tumour and normal cells. (B, C) Western Blot analysis for the hub genes in SW480 COCA cells, HT‐29 COCA cells and NCM460 normal endothelial cells. (D–F) AGRN expression was verified after transfection in AGRN. (G) Proliferation assays revealed attenuated capabilities of SW480 after knocking down AGRN. (H–J) Migration and invasion abilities of SW480 were weakened after knocking down AGRN. *p < 0.05, **p < 0.01, ***p<0.001, ****p<0.0001.

Researchers further validated the significance of AGRN in COCA by confirming the expression of four central genes in human normal colonic epithelial cells NCM460, as well as in the COCA cell lines HT‐29 and NCM460. Results indicated an upregulation of AGRN expression in HT‐29 and SW480, while MCF‐7 exhibited a significant variance (*p* < 0.05) (Figure 7A). The results from WB showed that HT‐29 and SW480 exhibited significantly higher expression levels compared to normal colonic epithelial cells (Figure 7B,C).

### 3.10 AGRN enhances the growth, movement and infiltration of COCA cells

The dysregulation of AGRN in COCA indicated a potential impact of AGRN on the development of COCA. In order to examine this theory, we effectively generated SW480 cell lines with reduced AGRN levels through the transfection of siRNA (Figure 7D,E). The proliferation capabilities of COCA cells were evaluated using CCK‐8 assays. Figure 7G demonstrates a notable decrease in the proliferation rate of SW480 cells upon AGRN knockdown. Cell migration and invasion abilities were evaluated using Transwell assays. It was obvious that migration and invasion capabilities were suppressed in AGRN knocked‐down cells (Figure 7H–J).

### 3.11 Analysis and Identification of hsa‐miR‐1307‐3p as Upstream Regulators of AGRN

We identified possible up‐regulated microRNAs associated with AGRN after confirming that AGRN expression is increased in COCA samples. We searched for miRNAs upstream of AGRN in starBase Version3.0. miRNA expression of COCA samples in TCGA were downloaded for a differential expression analysis. In total, 51 upregulated and 45 downregulated ncRNAs were obtained (*p* < 0.05, log2|FC| ≥ 2), and the volcano plot is shown in (Figure S1A). The intersection of DEmiRNAs with upstream microRNAs revealed 9 core downstream regulators (Figure S1B), including hsa‐miR‐15b‐5p, hsa‐miR‐149‐5p, hsa‐miR‐378a‐3p, hsa‐miR‐328‐3p, hsa‐miR‐331‐3p, hsa‐miR‐324‐3p, hsa‐miR‐339‐5p, hsa‐miR‐744‐5p, and hsa‐miR‐1307‐3p. Among the intersecting genes, hsa‐miR‐1307‐3p not only has a large area under the ROC curve which signified a high diagnostic accuracy (Figure S1C), but also is the only miRNA that has meaningful associations with patient prognosis (Figure S1D). These findings suggested that hsa‐miR‐1307‐3p is a hub miRNA in COCA and might downregulated AGRN expression.

## 4 DISCUSSION

Basement membranes (BMs) represent the most ancient form of animal extracellular matrix (ECM), forming sheet‐like structures that provide support to epithelial cells and envelop diverse tissues. Laminin and type IV collagen molecules organize into distinct planar networks that engage with cell surface interactors (CSIs) to establish the structural foundation of basement membranes within tissues. Furthermore, these molecules serve as signalling cues that direct cellular orientation, differentiation, motility and survival.^13^, ^14^, ^15^, ^16^ Variants in more than 20 BM genes have been implicated in the pathogenesis of human diseases,^17^ underscoring their diverse and essential functions. Autoantibodies directed against BM proteins are implicated in immune disorders, while dysregulation of protein expression and turnover in the BM is implicated in the development of cancer, diabetes, and fibrosis.^18^, ^19^, ^20^, ^21^ Through in silico prediction, over 1000 potential extracellular matrix (ECM) and ECM‐related proteins have been identified in vertebrates, with more than 700 in the model organism Caenorhabditis elegans.^21^, ^22^, ^23^, ^24^ BMs have diverse compositions tailored to resist mechanical stress, dictate tissue shape, and create diffusion barriers.^3^, ^14^, ^25^, ^26^ The preliminary analysis in this research involved the examination of 108 biomarkers exhibiting differing levels of expression in COCA tissues compared to normal tissues in the GTEx and TCGA databases. A systematic exploration of the biological pathways was conducted, and protein–protein interaction networks were constructed for the 108 biomarkers. Subsequently, 12 genes related to BMs and associated with COCA prognosis were identified through univariable and lasso‐penalized Cox regression analyses. Utilizing these 12 genes, a risk model was developed and validated in relation to the outcomes. The model exhibited robust predictive abilities as evidenced by survival and ROC analyses. Results from both single‐variable and multiple‐variable cox analyses indicated that the risk score generated from 12 clinical variables served as an independent prognostic factor for patients with COCA. Furthermore, a significant association between the signature and immune cell infiltration was observed, resulting in the identification of eight potential small‐molecule drugs for the treatment of COCA patients.

Analysis of the TIMER database revealed that 12 prognostic genes implicated in BMs are associated with immune cell infiltration, suggesting a potential role in cancer progression through immune modulation. The expression level of AGRN in genes related to BMs significantly correlates with patient prognosis, indicating its utility as a diagnostic marker. Subsequent AGRN knockout experiments in a laboratory setting demonstrated a marked reduction in tumour proliferation, migration and invasion capabilities. In conclusion, we obtained the BM‐related gene AGRN, which has a key pathogenic role for COCA and is also related to immune infiltration, drug target for anti‐leishmanial chemotherapy. Our studies demonstrate that AGRN drives COCA cells proliferation, migration and invasion abilities ex vivo.

This is the first research to detail the possible underlying mechanism of BMs in colorectal cancer and identify the key pathogenic gene—AGRN. The proteoglycan AGRN plays a crucial role in multiple signalling pathways, including those related to heart regeneration, synaptic differentiation and immune response.^27^, ^28^, ^29^ Its involvement in various human cancers, particularly lung adenocarcinoma and thyroid cancer, suggests its potential as a therapeutic target. AGRN's ability to enhance lung adenocarcinoma progression by activating the Notch signalling pathway and promote the tumorigenic phenotype of thyroid cancer cells, as well as its association with immune infiltration in thyroid tumours, underscores its significance in cancer biology.^30^, ^31^, ^32^ The upregulation of AGRN in rectal cancer has been shown to potentially facilitate disease progression by activating the WNT pathway. Matrine has been found to impede the advancement of colorectal cancer by modulating the AGRN/Wnt/β‐catenin pathway. Matrine impedes the advancement of colorectal cancer by modulating the AGRN/Wnt/β‐catenin pathway.^33^, ^34^ Approximately 30% of colorectal cancers originate from sessile serrated lesions (SSL), presenting a diagnostic challenge in distinguishing them from other polyp types. AGRN immunostaining, specifically targeting the muscularis mucosa, may serve as a novel biomarker for distinguishing SSL, hyperplastic polyps (HP), traditional serrated adenomas (TSA) and tubular adenomas (TA).^35^

The results of GSEA indicated that the signature derived from colorectal cancer (CR) was primarily associated with pathways related to cancer and metabolism, specifically the Protein digestion and absorption pathway. Therefore, the signature obtained from BMs can be used to predict the prognosis of patients with colorectal cancer (COCA) and play a significant role in the biological processes of COCA. Patients in high‐risk groups with COCA exhibited elevated expression levels of ADORA2A, CD276, ICOSLG, LAG3, LGALS9, PDCD1, TNFRSF14, TNFSF9 and VTCN1 compared to those in low‐risk groups, suggesting that the unfavourable prognosis observed in high‐risk COCA patients may be attributed to an immunosuppressive microenvironment. Moreover, individuals with COCA who are deemed high‐risk may derive benefits from the use of checkpoint inhibitor immunotherapies. Additionally, research has indicated that high‐risk individuals with COCA may experience improvements from treatments involving ondansetron, anabasine, dimethylprostaglandin E2, idoxuridine, fluvastatin, piribedil and trichlormethiazide.

miRNAs are critical regulators involved in the pathology mechanism of cancers, and thus serve as potential diagnostic markers and therapeutic targets.^36^, ^37^, ^38^, ^39^ The miRNA analysis of COCA showed that the upstream miRNA of AGRN might be miR‐1307‐3p. Previous studies have shown that miR‐1307‐3p expression is closely related to cancer development. miR‐1307‐3p has a significant contribution to the development and progression of breast cancer. Inhibition of miR‐1307‐3p may be a new strategy to inhibit the occurrence and progression of breast cancer. Qiu et al. suggested that miR‐1307 promoted the proliferation of prostate cancer by targeting FOXO3A. Simultaneous knockdown of miR‐1307 and FOXO3A can promote cell proliferation in prostate cancer. miR‐1307 is an important anti‐apoptotic oncoprotein, known to promote chemotherapy resistance in ovarian cancer by targeting ING5 expression, and miR‐1307 may be a therapeutic target for ovarian cancer.^40^

The study is subject to certain limitations. Experimental validation is required to ascertain the mechanisms by which basement membranes regulate the biological functions of COCA cells. Additionally, the viability of the prognostic model must be substantiated through a multicenter clinical trial.

In conclusion, we identified differentially expressed BMs that might be involved in the progression of COCA. AGRN has important values in predicting the outcome of COCA patients and targeting AGRN showed the potential application as an effective treatment of COCA. Our study also should be validated by further research.

## AUTHOR CONTRIBUTIONS

**Jianrong Li:** Methodology (equal); validation (equal). **Daofeng You:** Data curation (equal). **Linjie Hu:** Methodology (equal); validation (equal). **Yusi Yang:** Data curation (equal). **Sheng Gao:** Resources (equal). **Wenqi Bai:** Funding acquisition (equal).

## FUNDING INFORMATION

Shanxi Province Cancer Hospital/ Shanxi Hospital Affiliated to Cancer Hospital, Chinese Academy of Medical Sciences, Fund to guide and accompany flying master students (SD2023010).

## CONFLICT OF INTEREST STATEMENT

The authors confirm that there are no conflicts of interest.

## Supporting information


Figure S1.

**File S1.** The flow chart of the study.

**File S2.** The result of the difference analysis.

## Data Availability

All files are provided in the supplementary file in the article.
