# Supplementary material for: Identification and validation of basement membrane‐associated gene AGRN as prognostic and immune‐associated biomarkers in colorectal cancer patients
Source: J Cell Mol Med. 2024 Aug 25;28(16):e70010. doi: 10.1111/jcmm.70010 (PMC11345205; doi:10.1111/jcmm.70010)
Supplement: Supplementary file 1 — Figure S1. [file JCMM-28-e70010-s002.pdf]

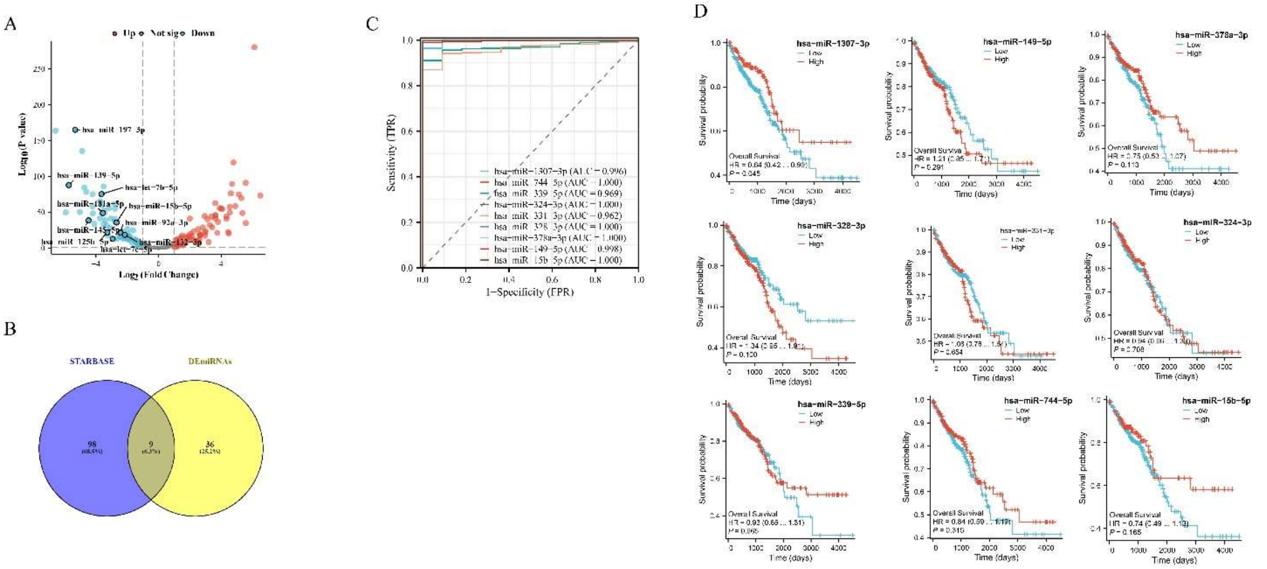

Supplementary Figure 1 Identification of AGRN upstream miRNAs associated with COCA. (A) Volcano plot of the DEmiRNAs. Red, upregulation; blue, downregulation. (B) Intersection of DEmiRNAs and upstream miRNAs of AGRN. (C) ROC analysis of intersection miRNAs. (D) The overall survival curves of intersection DEmiRNAs.
