# Supplementary figures and images for: Identification and validation of basement membrane‐associated gene AGRN as prognostic and immune‐associated biomarkers in colorectal cancer patients
Source: J Cell Mol Med. 2024 Aug 25;28(16):e70010. doi: 10.1111/jcmm.70010 (PMC11345205; doi:10.1111/jcmm.70010)

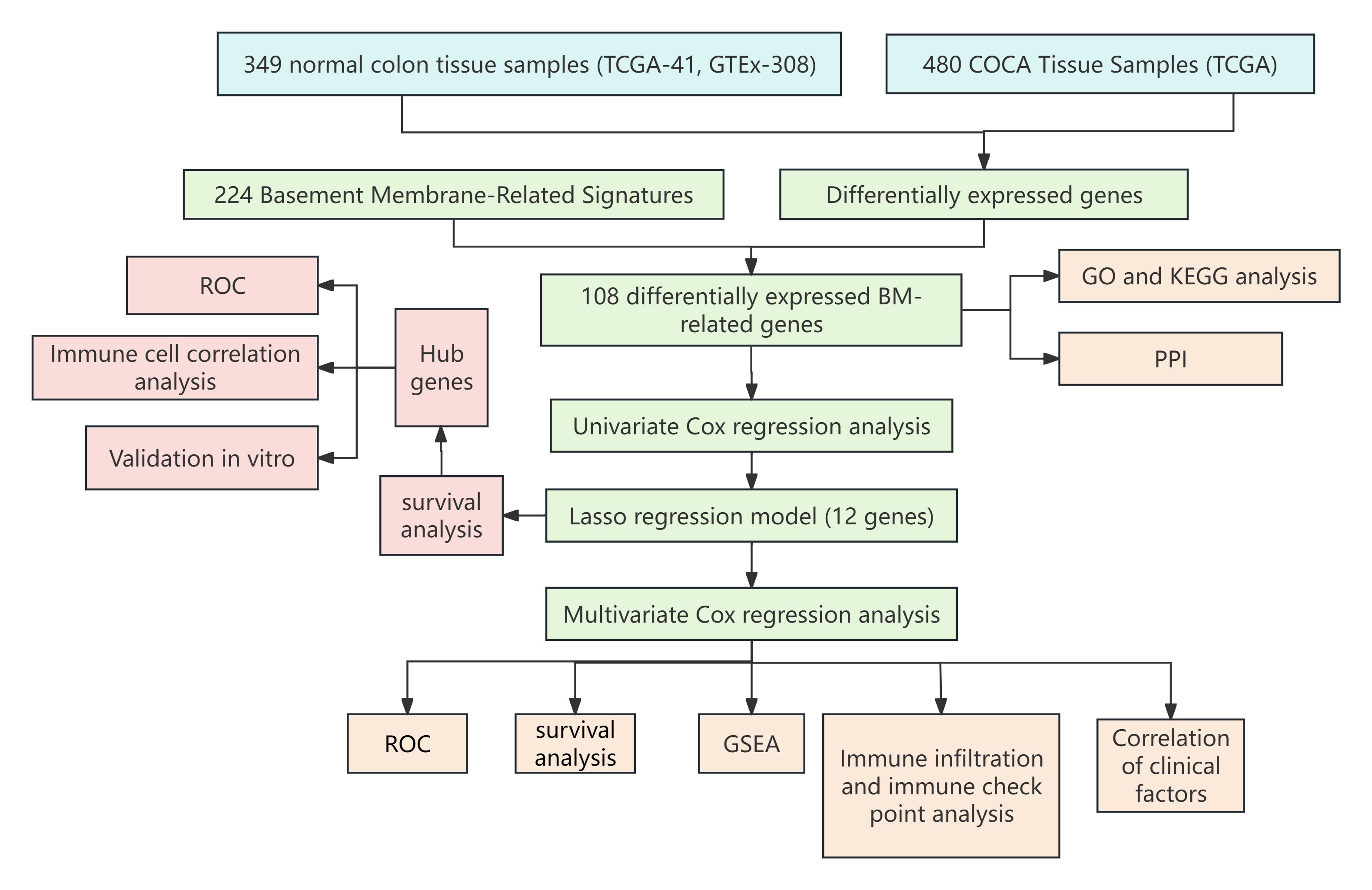

Supplement: Supplementary file 2 — File S1. The flow chart of the study. [file JCMM-28-e70010-s001.jpg]
